# Bacterial attachment and junctional transport function in induced apical-out polarized and differentiated canine intestinal organoids

**DOI:** 10.3389/fvets.2024.1483421

**Published:** 2024-12-18

**Authors:** Shino Yoshida, Meg Nakazawa, Minae Kawasaki, Yoko M. Ambrosini

**Affiliations:** Department of Veterinary Clinical Sciences, College of Veterinary Medicine, Washington State University, Pullman, WA, United States

**Keywords:** organoid, canine, polarity reversal, apical-out, intestine, differentiation, host-pathogen interaction

## Abstract

Dogs are increasingly recognized as valuable large animal models for understanding human intestinal diseases, as they naturally develop conditions similar to those in humans, such as Enterohemorrhagic *E. coli*, *Clostridium difficile infection*, inflammatory bowel disease, and ulcerative colitis. Given the similarity in gut flora between dogs and humans, canine *in vitro* intestinal models are ideal for translational research. However, conventional extracellular matrix-embedded organoids present challenges in accessing the lumen, which is critical for gut function. This study aimed to investigate the feasibility of inducing polarity reversal and differentiation in canine apical-out colonic organoids (colonoids), evaluate their barrier integrity, and visualize host-pathogen interactions. Our results demonstrated successful polarity reversal and differentiation induction while maintaining barrier integrity. Polarity reversal allowed for enhanced observation of host-pathogen interactions, facilitating visual assessments and membrane integrity evaluations using both pathogenic and nonpathogenic *E. coli*. This process led to the downregulation of stem cell marker *LGR5* and upregulation of intestinal epithelial cell marker *ALPI*, indicating differentiation. Further differentiation was observed with the use of a differentiation culture medium, resulting in significant upregulation of *ALPI* and goblet cell marker *MUC2*. The findings suggest that apical-out canine colonoids can serve as physiologic and valuable models for studying the pathogenic mechanisms and clinical significance of intestinal diseases in dogs. This model has the potential to advance both canine and human gastrointestinal research, enhancing our understanding of gastrointestinal physiology and pathology and aiding in the development of novel therapeutics.

## 1 Introduction

Dogs are recognized as important large animal models for understanding human intestinal disease processes, as dogs naturally develop similar chronic intestinal diseases as humans, such as Enterohemorrhagic *E. coli* (EHEC) (1, 2), *Clostridium difficile* infection (3, 4), inflammatory bowel disease (IBD) and ulcerative colitis (5).

Organoids are promising *in vitro* models that can be established from isolated stem cells of the intestinal tissue biopsies and have been shown to consist of variable populations similar to intestines *in vivo* (6). The one flaw for conventional extracellular matrix-embedded organoids is the difficulty in accessing their lumen, which plays a vital role in gut function. Polarity reversal is one technique enabling access to the apical border (7). The possibility of establishment and application of these apical-out enteroids are investigated in various animals, such as cattle (8), porcine (9, 10), and dogs (11). However, there is contradicting evidence on their characteristics, which might be attributed to the differences in species, protocol, and culture medium composition. A previous study that induced polarity reversal in canine intestinal organoids failed to show gene expression differentiation (11). In extracellular matrix-embedded intestinal organoids, multi-lineage differentiation was observed using a differentiation culture medium (DM) (12, 13). Therefore, the use of the differentiation culture medium in polarity-reversed intestinal organoids possibly induces multi-lineage differentiation as well. Moreover, as barrier integrity is essential for maintaining a healthy gut environment (14), the effect of the apical-out procedure should be investigated. Furthermore, the exposure of its apical border will enable more physiological replication of host-pathogen interactions, leading to further application to understand the gastrointestinal disease process.

Therefore, this study aims to investigate the feasibility of differentiation induction in canine apical-out colonic organoids (colonoids), evaluate their barrier integrity, and visualize the host-pathogen interaction with infectious agents.

## 2 Materials and methods

### 2.1 Canine biopsy derived colonoid culture

In this study, intestinal biopsies were derived from healthy donors anesthetized for dental cleaning procedures. The donor population of 1- to 12-year-old dogs with no history of chronic diseases affecting the gastrointestinal tract, heart, kidney, and liver was recruited. These dogs were comprehensively screened with physical and blood examinations as pre-anesthetic evaluations and deemed healthy other than dental diseases. This study was conducted with the approval of the Washington State University Institutional Animal Care and Use Committee (IACUC Approval: ASAF#6993). Colonoids were established and maintained as previously described (15). Briefly, colonoids were embedded in 30 μL of Matrigel (Corning, New York, USA) on 48-well plates (Corning), incubated at 37°C for 10 min, and added with 300 μL of expansion medium. The basal medium consisted of Advanced Dulbecco’s Modified Eagle Medium (DMEM) /F12, 2 mM of Glutamax-I, 10 mM of N-2-hydroxyethyl piperazine-N-2-ethane sulfonic acid, 1x Penicillin/streptomycin. The composition of the expansion medium was as follows; basal medium, 1x B27 supplement (provided from Gibco, Thermofisher Scientific, Massachusetts, USA), 10% (vol/vol) of Noggin Conditioned Medium, 20% (vol/vol) of R-Spondin-1 Conditioned Medium (made in the laboratory by culturing HEK293 cells), 100 ng/mL of Recombinant Murine Wnt-3a, 50 ng/mL of murine Epidermal Growth Factor, 100 μg/mL of Primocin (derived from PeproTech, Thermofisher Scientific, Massachusetts, USA), 10 nM of gastrin I human, 500 nM of A 83–01, 10 μM of SB202190, 10 mM of Nicotinamide (Sigma Aldrich, Missouri, USA), 1 mM of N-Acetyl-L-Cysteine (MP Biomedicals, Southern California, USA), 1x N2 Max Media supplement (R&D Systems, Minnesota, USA). For the first 2 days after passaging, 10 μM of Y-27632 and 2.5 μM of CHIR 99021 (Stem Cell Technologies, Vancouver, Canada) were added to the medium.

### 2.2 Polarity reversal and differentiation of colonoids

**Figure 1:**
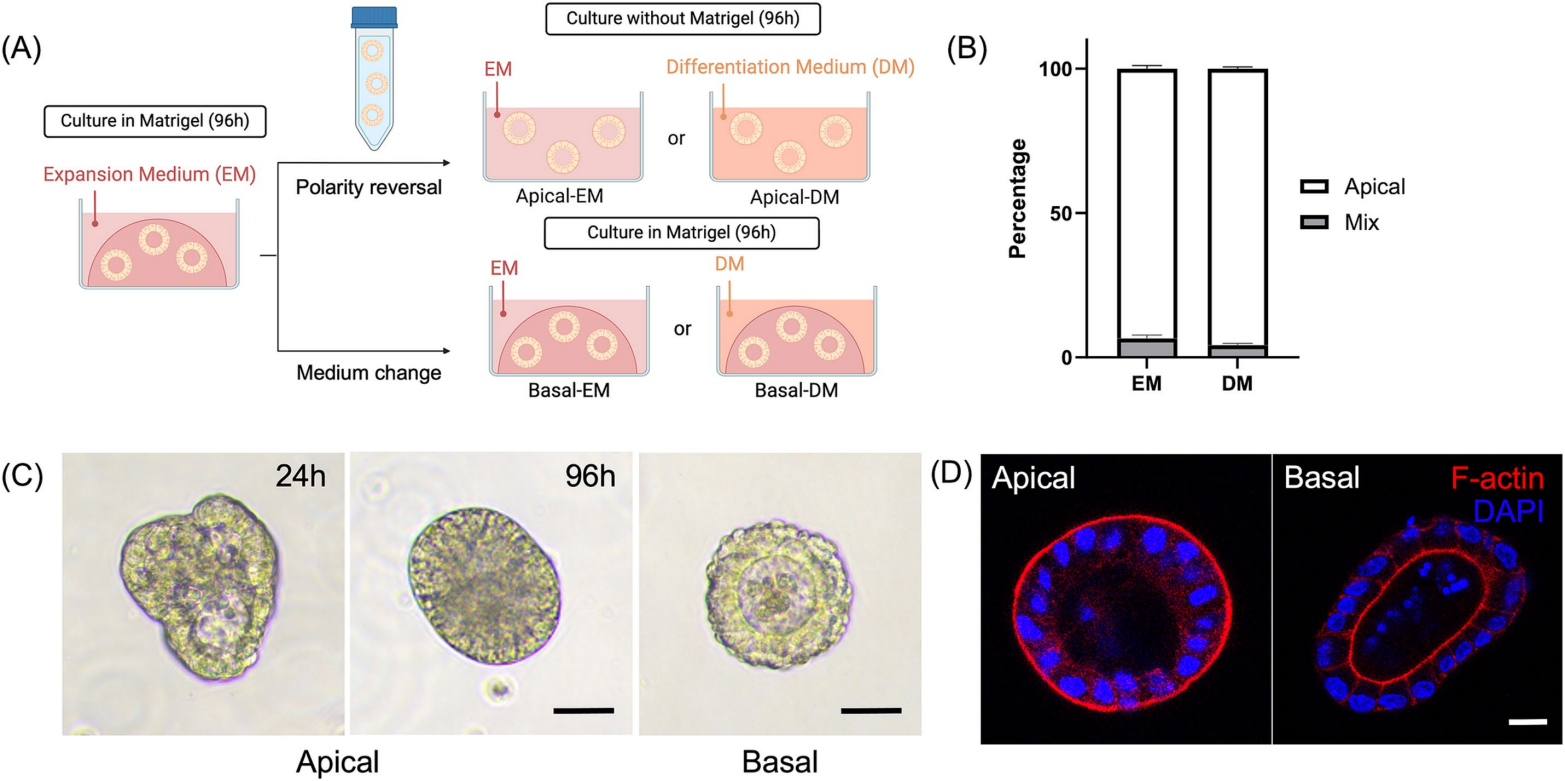
Development and characterization of the canine apical-out colonoids. **(A)** A schematic for polarity reversal of colonoids. **(B)** The percentage of apical-out colonoids (Apical, white box) and colonoids with mixed polarities (Mix, gray box) under confocal microscopy at 96 h (*n* = 3, each). Data is shown as mean ± standard error of the mean (SEM). 40–60 randomly selected organoids in each biological replicate were counted and classified as apical-out, basal-out, or mixed polarity. **(C)** Representative images from the phase-contrast microscope at 24 and 96 h. Scale bar = 25 μm. **(D)** Confocal microscopy images of an apical-out colonoid and Matrigel-embedded colonoid at 96 h with visualization of apical brush borders (F-actin, red) and basal nuclei (DAPI, blue). Scale bar = 10 μm.

Apical-out canine colonoids were established according to the previous reports that established apical-out organoids from the human intestine (16). Briefly, the colonoids were harvested 4 days after passage, suspended and cultured in 5 mM of ethylenediaminetetraacetic acid (EDTA)-phosphate-buffered saline (PBS) for an hour on a rotating platform, centrifuged and removed EDTA, washed with the basal medium, and resuspended to the 400 μL of desired medium to a 24-well ultra-low-attachment plate (Corning, New York, USA). One well of Matrigel-embedded colonoids was suspended to three wells of floating colonoids. The number of colonoids was counted and confirmed as not exceeding 500 colonoids/well. Twelve hours after resuspension, floating colonoids were dislodged by pipetting to prevent sticking to each other. To clarify the effect of the different culture conditions on gene expression, apical-out colonoids were cultured either in the expansion medium (EM) or differentiation medium (DM) (Figure 1A). According to the previous report, DM was prepared as an EM without Wnt-3a, nicotinamide, and SB202190 (12). The medium was changed every other day.

### 2.3 Immunofluorescent staining

Colonoids were fixed with 4% paraformaldehyde (PFA) for 30 min at room temperature and washed with PBS. The sample was permeabilized with 0.3% Triton™ X-100 (Sigma Aldrich, Missouri, USA) in PBS for 10 min at room temperature and washed with PBS. The sample was incubated with 2% bovine serum albumin (BSA) for an hour to prevent unspecific binding of antibodies. When indicated, colonoids were incubated with 1:50 anti-chromogranin A antibody (ab45179, Abcam, Cambridge, United Kingdom) for enteroendocrine cells and 1:100 *Sambucus nigra* lectin; SNA (Vector Laboratories, California, USA) for mucus overnight at 4°C, washed with PBS, and then treated with 1:1000 Alexa Fluor 555-conjugated Goat Anti-Rabbit IgG H&L secondary antibody (Abcam) for an hour at room temperature, followed by another wash with PBS. Colonoids were incubated with 1:400 phalloidin (Alexa Fluor™ 647 Phalloidin) for actin filaments and 1:1000 diamidino-2-phenylindole (DAPI) for nuclear staining (Invitrogen, Thermo Fisher Scientific, Massachusetts, USA) for 30 min at room temperature and washed. Colonoids were suspended in ProLong™ Gold Antifade Mountant (Invitrogen) onto the Glass Bottom Culture Dishes (Matsunami Glass, Osaka, Japan) and imaged using TCS SP8 X White Light Laser Confocal Microscope (Leica, Wetzlar, Germany). Images were processed with LAS X software (Leica). The number of polarity reversed cells was counted 96 h after the polarity reversal under the confocal microscope and considered apical-out if F-actin was aligned outside and mixed if F-actin alignment was observed both outside and inside. The percentage of staining positive cells was calculated by dividing the number of positive cells by the total number of nuclei. This experiment was conducted using three biological replicates.

### 2.4 Reverse transcription-quantitative polymerase chain reaction

After 96 h of culture in EM or DM, colonoids were collected and washed with PBS. A time point of 96 h was chosen based on previous reports (13, 17). Total RNA was isolated using an RNeasy Mini Kit (Qiagen, Hilden, Germany). cDNA was synthesized using the High-Capacity cDNA Reverse Transcription Kit (Applied Biosystems, Thermo Fisher Scientific, Waltham, Massachusetts, USA) with the C1000 Touch Thermal Cycler (Bio-Rad Laboratories, California, USA). The quantitative polymerase chain reaction (qPCR) was conducted using PowerUp SYBR Green Master Mix (Applied Biosystems) with the CFX96 Touch Real-Time PCR Detection System (Bio-Rad Laboratories). Gene expression levels of the following marker genes were evaluated: leucine-rich repeat-containing G protein-coupled receptor 5 (*LGR5*), intestinal alkaline phosphatase (*ALPI*), mucin 2 (*MUC2*), and chromogranin A (*CHGA*). Hydroxymethyl-bilane synthase (*HMBS*) and Succinate dehydrogenase complex subunit A (*SDHA*) were selected as internal references (18). The relative quantity of each gene was calculated using the standard curve method and normalized with the internal reference genes as previously described (19). The primers used in this study are listed in Supplementary Table S2 (13, 18, 20, 21). This experiment was conducted using three biological replicates and three technological replicates, and each sample was examined in duplicate for qPCR, and the mean values were used for analysis.

### 2.5 Dextran diffusion barrier integrity assay

The barrier integrity was assessed in accordance with the previous report (16). After 96 h of polarity reversal and subsequent culture in DM, colonoids were collected and pelleted at 300 *g* for 1 min. To make the barrier-disrupted model a positive control, apical-out colonoids were treated with 2 mM EDTA for 15 min on ice. After treatment, colonoids were washed with basal medium and resuspended to 100 μL of 2 mg/mL 4 kDa fluorescein isothiocyanate (FITC)-dextran (Sigma-Aldrich). After 5 min, 90 μL of FITC-dextran supernatant was removed, and 10 μL of the remaining bottom FITC-dextran was transferred to a well marked using a PAP pen on a glass microscope slide. To avoid crushing colonoids when putting the coverslip, grease spots were loaded on the slide in a way consistent with the four corners of the coverslip. After preparation, confocal images were immediately obtained using the EVOS FL Fluorescence Microscope (Advanced Microscopy Group, Washington, USA). FITC intensity of colonoids was measured by tracing its outline and normalized divided by intensity outside in order to reduce background discrepancies and enable more accurate comparison of FITC intensities across samples [three data points averaged; (22)] using ImageJ version 2.14.0. Therefore, the low FITC permeability ratio means low permeability. This experiment was conducted using three biological replicates.

### 2.6 *Escherichia coli* infection

A wild strain of Enterohemorrhagic *Escherichia coli* O157:H7 (EHEC) derived from bovine feces was used as clinical isolates of canine were unavailable (23). A nonpathogenic strain of *Escherichia coli* (*E. coli*) tagged with YFP, MC4100, was used as an *E. coli* infection control, which was generously provided by Dr. Roy Kishony (Harvard Medical School, Massachusetts, USA). *E.coli* were incubated overnight in Luria-Bertani broth at 37°C on shaking at 200 rpm, then diluted 1:10 and subcultured for 1.5 h. Bacteria were harvested, washed with PBS, and resuspended to 1.0 × 10^8^ CFU/mL in DM without antibiotics. After 96 h of polarity reversal and subsequent culture in DM, colonoids were harvested and washed with basal medium without antibiotics, resuspended to the bacteria-containing DM, and incubated at 37°C for 4 h. Before immunofluorescence staining and the FITC-dextran assay, colonoids were washed with PBS once. This experiment used three biological replicates.

### 2.7 Statistical analysis

The gene expression levels between apical-out colonoids and Matrigel-embedded basal-out colonoids and within apical-out colonoids cultured in different mediums (EM or DM) were compared using the Willcoxon rank-sum test, followed by Bonferroni correction. Results were shown as mean ± standard error of the mean (SEM). The FITC-dextran assay data was also compared using the Willcoxon rank-sum test, followed by Bonferroni correction when comparing three groups. *p* < 0.05 were considered statistically significant differences. Graphs were produced using Prism (10.2.1) (GraphPad Software, San Diego, California, USA), and statistical analysis was conducted using R version 4.0.2.

## 3 Results

### 3.1 Development and characterization of the canine apical-out colonoids

Colonoids cultured in a suspension with an ultra-low-attachment plate (Figure 1A) showed morphological changes. The percentage of the completely polarity-reversed colonoids was counted 96 h after the polarity reversal using the confocal microscope. Over 93% of colonoids were counted as apical-out colonoids in both EM and DM (Figure 1B). Under phase-contrast microscopy, the cell borders of columnar cells were more prominent, and these colonoids lacked an apparent lumen, contrary to the Matrigel-embedded colonoids with an obvious central lumen. The change started as early as 24 h following the 5 mM EDTA/PBS treatment (Figure 1C). Immunofluorescence staining demonstrated that F-actin was expressed on the outer surface, which confirmed the successful polarity-reversal of canine colonoids (Figure 1D).

### 3.2 Gene expression analysis and immunocytochemistry

**Figure 2:**
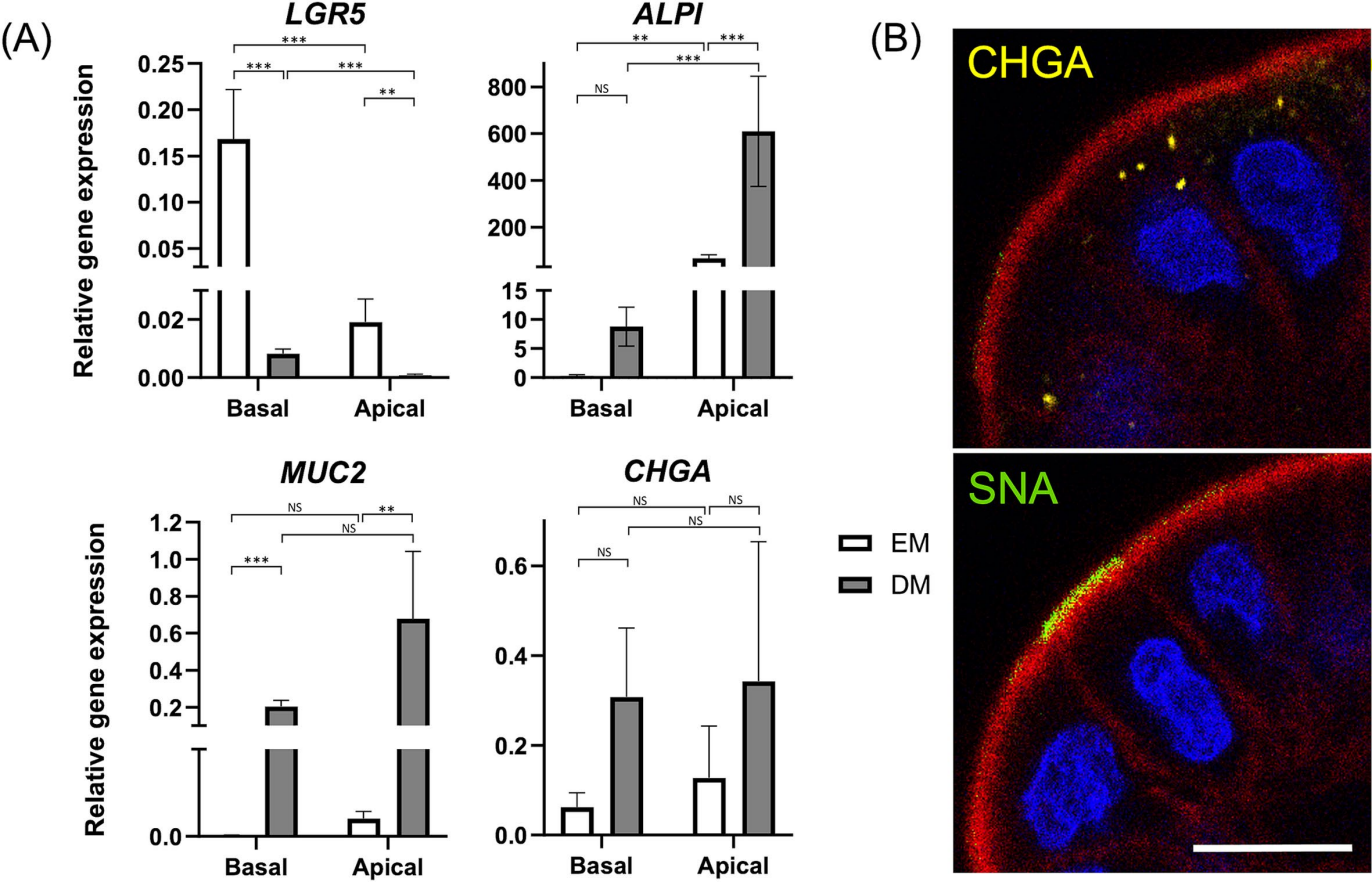
Characterization of apical-out colonoids. **(A)** Comparison of relative gene expression levels of the following markers: *LGR5* (stem cells), *ALPI* (Intestinal epithelial cells), *MUC2* (goblet cells), and *CHGA* (enteroendocrine cells) of apical-out colonoids in EM (white box) versus DM (gray box). Data is shown as mean ± SEM. *p* < 0.05 was considered statistically significant using the Willcoxon rank-sum test, followed by Bonferroni correction. *p < 0.05; **p < 0.01, ***p < 0.001. **(B)** Confocal microscopy images of apical-out colonoids at 96 h in DM with visualization of apical brush borders (F-actin, red) and basal nuclei (DAPI, blue), chromogranin A (CHGA, yellow), and *Sambucus nigra* lectin (SNA, green). Scale bar = 10 μm.

To investigate the effect of differentiations by polarity reversal, gene expression levels of the following marker genes were evaluated between Apical-out colonoids (Apical) and basal-out colonoids (Basal): *LGR5* (stem cells), *ALPI* (absorptive enterocytes), *MUC2* (goblet cells), and *CHGA* (enteroendocrine cells) (Figure 2A). In Apical, *LGR5* was significantly downregulated (*p* < 0.001 both in EM and DM), and *ALPI* was significantly upregulated (*p* = 0.002 in EM, *p* < 0.001 in DM).

Furthermore, to clarify the effect of differentiations by the medium, these gene expressions were compared between Apical-out colonoids cultured in EM (Apical-EM) and Apical-out colonoids cultured in DM (Apical-DM). Apical-DM showed further downregulation of *LGR5* (*p* = 0.002) and upregulation of *ALPI* (*p* < 0.001) compared to Apical-EM. In addition, upregulation of *MUC2* was observed in Apical-DM (*p* = 0.005). A significant difference was not observed in *CHGA* in either comparison. Immunofluorescence of *Sambucus nigra* lectin (SNA) and CHGA revealed the goblet cell and enteroendocrine cell differentiation of Apical (Figure 2B). The percentage of CHGA-positive and SNA-positive cells were 31.5 ± 6.80 and 33.6 ± 7.64%, respectively (Supplementary Figure S1).

### 3.3 Dextran diffusion barrier integrity assay

**Figure 3:**
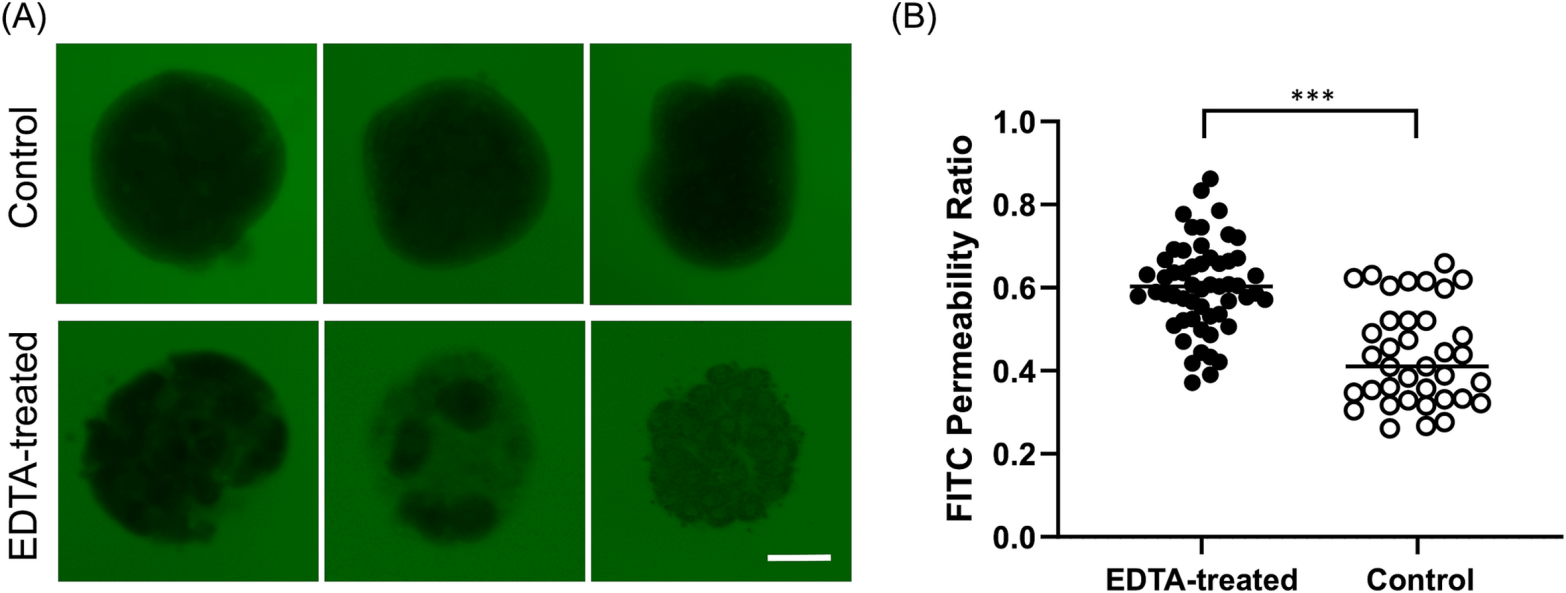
The evaluation of barrier integrity using FITC-dextran assay at 96 h. **(A)** Representative image of untreated Apical-DM and EDTA-treated Apical-DM. Scale bar = 50 μm. **(B)** FITC permeability ratio of untreated (Control, white dots) and EDTA-treated Apical-DM (black dots). The median is shown as a horizontal line. For image acquisition, randomly selected 6–15 fields were used in each biological replicate. *p* < 0.05 was considered statistically significant using the Willcoxon rank-sum test. ***p < 0.001.

A barrier integrity assay was conducted to assess whether Apical-DM maintained functional tight junctions. EDTA-treated Apical-DM served as a model for disrupted barrier function. Untreated Apical-DM (Control) maintained its spherical shape with a clear outline, with no fluorescent penetration observed in the control group, unlike the EDTA-treated group (Figure 3A). The FITC permeability ratio was significantly lower in the control group compared to the EDTA-treated Apical-DM (*p* < 0.001), indicating that untreated colonoids maintained their barrier integrity (Figure 3B). Immunofluorescence images of the control and EDTA-treated group are shown in Supplementary Figure S2, and disruption of the membrane was observed in the EDTA-treated group.

### 3.4 Host–pathogen interaction

**Figure 4:**
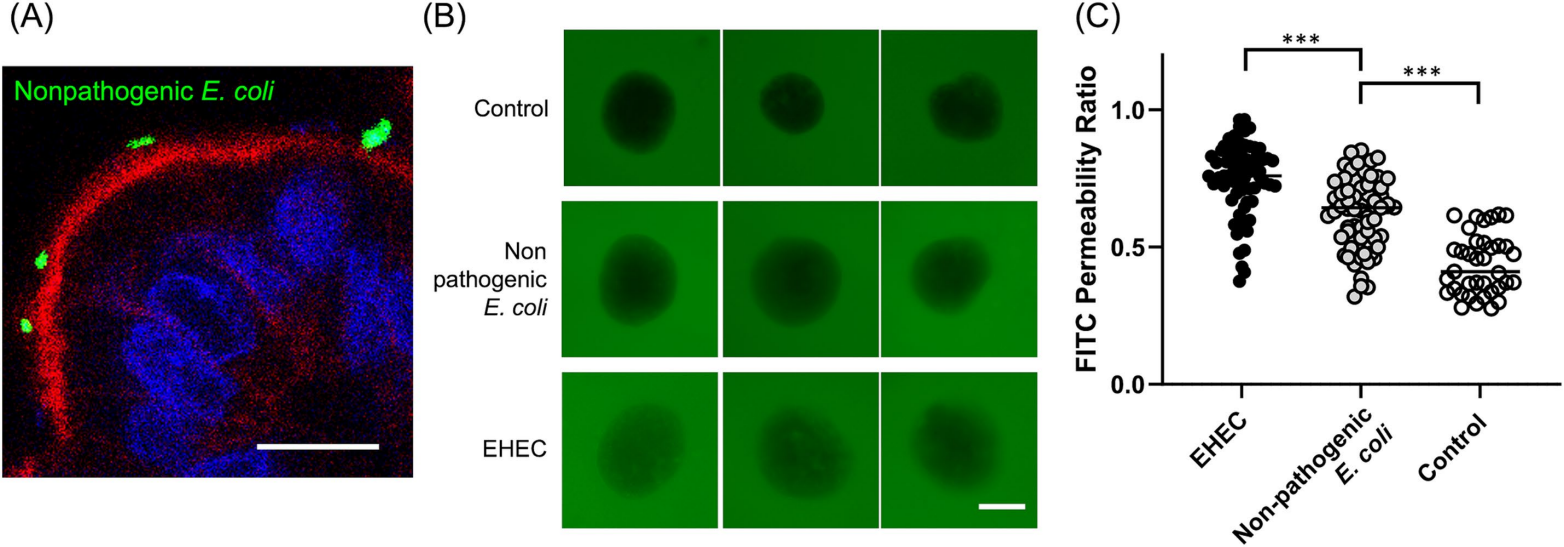
Host-pathogen interaction. **(A)** A representative confocal microscopy image of apical-DM infected with nonpathogenic *E. coli* (YFP-tagged, green) with visualization of apical brush borders (F-actin, red) and basal nuclei (DAPI, blue). Scale bar = 10 μm. **(B)** In barrier integrity assay, representative images of apical-out colonoids infected with nonpathogenic *E. coli* or EHEC. Scale bar = 50 μm. **(C)** FITC permeability ratio of untreated (Control, white dots), apical-out colonoids infected with nonpathogenic *E. coli* (gray dots), or EHEC (black dots). The median is shown as a horizontal line. For image acquisition, randomly selected 6–12 fields were used in each biological replicate. *p* < 0.05 was considered statistically significant using the Willcoxon rank-sum test. ***p < 0.001.

The immunofluorescence image demonstrated nonpathogenic *E. coli* attachment to the apical surface (Figure 4A). A barrier integrity assay was further conducted to determine whether pathogenic EHEC infection for 4 h could cause infection and barrier disruption in Apical-DM. Representative images of Apical-DM infected with nonpathogenic *E.coli* or EHEC are shown in Figure 4B. EHEC-infected Apical-DM showed a significantly higher FITC permeability ratio compared to nonpathogenic *E. coli*-infected Apical-DM (*p* < 0.001; Figure 4C), demonstrating EHEC-induced barrier disruption in Apical-DM as reported previously in other models (23, 24). Nonpathogenic *E. coli* showed a higher FITC permeability ratio than the control (*p* < 0.001). Supplementary Figure S3 presents immunofluorescence images of control, nonpathogenic *E. coli*-infected, and EHEC-infected Apical-DM. Cell membrane disruption was observed exclusively in EHEC-infected samples, with DAPI nuclear staining showing free cells detached from the organoid structure (Supplementary Figure S3, white arrows).

## 4 Discussion

This study demonstrated successful polarity reversal and differentiation induction while maintaining barrier integrity. Additionally, the polarity reversal facilitated the observation of host-pathogen interactions, allowing for visual assessments and membrane integrity evaluations using both pathogenic and nonpathogenic *E. coli*.

In this study, polarity reversal resulted in the downregulation of stem cell markers *LGR5* and the upregulation of intestinal epithelial cell marker *ALPI*. This finding aligns with a previous report on porcine organoids, which showed significant downregulation of *LGR5* and upregulation of *ALPI* following polarity reversal (10). However, this trend was not observed in another study involving canine intestinal organoids (11). Similar to findings with basal-out intestinal organoids (25, 26), optimizing media conditions for apical-out organoids could effectively promote differentiation into specific intestinal epithelial cell lineages of interest. Compared to the previous study using canine apical-out intestinal organoids (11), the composition of the culture medium may have influenced the outcomes (Supplementary Table S3). Moreover, further successful cell differentiation with our differentiation medium was observed, marked by significant upregulation of *ALPI* and the goblet cell marker *MUC2*. As noted, the composition of the culture medium significantly impacts organoid differentiation (12). Our differentiation medium, formulated based on these findings, effectively induced enterocyte and goblet cell differentiation. Furthermore, the percentages of enteroendocrine cells (27) and goblet cells (28) were similar to previous publications in human and dog colons, which indicates that Apical-DM reproduces the *in vivo* cell populations. Our protocol for inducing further differentiation of apical-out colonoids will help us better mimic the physiological intestine, enhancing its role as an *in vitro* model (13, 29).

As shown in Figure 4A, the current study successfully visualized the attachment of *E.coli* to the apical surface of canine colonoids. The critical feature of bacterial infection is host-pathogen interaction with the intestinal epithelium, especially interaction with host actin, which has been proven using enteropathogenic and enterohemorrhagic *E. coli* (30). Reversing polarity to expose the apical surface enables more physiological replication of host-pathogen interactions, providing a model that better mimics natural infection processes. This approach can significantly enhance our understanding of the pathogenic mechanisms underlying infectious diseases.

We demonstrated that apical-out colonoids maintained their essential barrier function, which is characteristic of a healthy intestine. This barrier integrity was shown to be disrupted by the infection of a pathogenic strain of *E.coli*, which is consistent with the previous reports (31, 32). Notably, the FITC permeability ratio was higher in Apical-DM infected with nonpathogenic *E. coli* compared to the control group, indicating that nonpathogenic *E. coli* also induced barrier disruption, although its effect was less significant than that of EHEC. Similar findings were observed in a previous study with human intestinal organoids, where infection with a commensal strain of nonpathogenic *E. coli* resulted in reduced barrier function and reactive oxygen species production, albeit to a lesser extent than EHEC infection (33).

Dogs can naturally develop IBD, which is different from other potential model animals like pigs and mice (34). IBD is a complex disorder, and susceptibility genes, epithelial barrier function, immune responses, and commensal bacteria and pathogens seem to play roles in the development of IBD (35). Some pathogenic processes, such as having genetic variants of Nucleotide Oligomerization Domain Two (36, 37), single nucleotide polymorphism in major histocompatibility complex (38, 39), upregulation of Toll-like receptors 2 and 4 (40, 41) were observed in both human and canine IBD patients. Also, dog gut microbiome is closer to human gut microbiome than pigs and mice (42), and these microbiomes are also considered important in the development of IBD both in humans and dogs. These features make dogs a substantial, large animal model for translational research.

In this study, *E. coli* MC4100 was used as a nonpathogenic strain. MC4100 was constructed by transposing the *lacZ* genes to the *E. coli* K-12 strain (43). Originally isolated from a human convalescent diphtheria patient in 1922 (44), *E. coli* K-12 is a well-established benign laboratory strain devoid of known virulence factors (45, 46) and has been used across various species, including humans, mice, cattle, and birds (47–50). In canine colonoids, MC4100 showed distinct effects on barrier integrity compared to pathogenic EHEC (51) (Figure 4). While MC4100 has undergone genetic deletions compared to other K-12 derivatives (52, 53), no acquisition of virulence factors has been reported. Therefore, it was used to serve as a nonpathogenic control in this study.

EHEC has been shown to cause acute diarrhea in dogs, with watery or mucoid stools and vomiting, and can result in fatal hemorrhagic diarrhea in critically ill patients (2, 54). The clinical similarities between EHEC infections in humans and dogs (55) highlight the relevance of studying EHEC pathogenicity in canine models. In this study, we used a cattle-sourced EHEC isolate, recognizing that canine-derived isolates might produce different results. However, as cattle are the primary reservoirs of EHEC, bovine isolates have been implicated in canine outbreaks, particularly in dogs consuming raw meat. This route of transmission mirrors human infections, often linked to contaminated dairy or meat products (1, 56). Thus, we considered a bovine isolate to be a reasonable choice for modeling natural EHEC transmission in dogs.

Although EHEC is considered to be an important pathogen in dogs, epidemiological data indicated that Enteropathogenic *E. coli* (EPEC) is more commonly found in dogs than EHEC. For instance, a study showed the prevalence rate of EPEC was 41% in dogs with diarrhea and 6% in healthy household dogs, while EHEC probe-positive isolates were not found in this study (57). Also, cases of acute gastroenteritis of EPEC infection have been reported in dogs (58). This prompts the question of whether host-specific factors influence susceptibility to EHEC infection. Comparing gene expression responses in human and canine colonoids following EHEC infection could help elucidate why humans are generally more susceptible to EHEC, whereas dogs are more frequently affected by EPEC.

Segment-specific organoids are powerful tools for studying GI pathogens like EPEC and EHEC, as they replicate unique cellular and physiological traits of each intestinal region (59). For example, small intestinal organoids (enteroids) allow precise modeling of EPEC’s disruption of tight junctions and nutrient, water, and solute absorption, which leads to osmotic diarrhea (60). Conversely, large intestinal organoids (colonoids) enable studies of EHEC’s toxin-mediated damage to colonic epithelium and vasculature, leading to hemorrhagic colitis (61). This segmentation in organoid technology enables pathogen studies that closely mimic disease processes in specific gastrointestinal regions. Canine small intestinal (duodenal, jejunal, ileal) organoids have already been established (6) and are also stably utilized in our laboratory. Additionally, a previous study established jejunal apical-out enteroids (11), although gene multi-lineage differentiation was not observed. In human basal-out enteroids, differentiation medium without Wnt3a, nicotinamide, and SB202190 also led to multi-lineage differentiation (62), similar to colonoids (12). Using the same methodology in the current study, canine apical-out enteroids with multi-lineage differentiation could be established, accelerating the understanding of the segment-specific pathophysiology of pathogens and distinct host-pathogen interactions that are specific or similar between hosts and species.

This study did not include gene expression analysis following *E. coli* infection. Different species and pathogens exhibit unique molecular mechanisms of pathogenicity (63, 64). In human intestinal organoids, gene expression analysis has shown pathogen-specific inflammatory responses. For example, EHEC-infected colonoids show upregulated inflammatory genes, including IL-8, compared to non-pathogenic *E. coli*-infected organoids (33). Conversely, *Salmonella* strains induce distinct responses: *S. typhi* downregulates pro-inflammatory signaling, *S. typhimurium* suppresses cell cycle and metabolic pathways, while *S. enteritidis* upregulates them (65). We anticipate that future studies with canine apical-out organoids will similarly demonstrate pathogen- and species-specific host-pathogen interactions. This study established apical-out colonoids with multi-lineage differentiation. These apical-out intestinal organoids are excellent models for infectious diseases and subsequent gene expression analysis, and they would have laid the foundation for future studies to accelerate our understanding of the molecular mechanism in infectious diseases.

Another culture method to address the limitation of conventional basal-out organoids is organoid-derived monolayer culture. This method has several advantages. It enables the access and manipulation of both apical and basal surfaces and a better assessment of epithelial barrier function and permeability (66). However, developing a monolayer is labor-intensive; it requires multiple steps and time to establish, dissociating organoids to single cells and seeding onto culture inserts. It requires several days for maturation. Therefore, apical-out organoids are considered less complex, alternative methods to access the apical side.

One limitation of this study is that intestinal membrane integrity was assessed indirectly using the FITC-dextran assay. The FITC permeability ratio quantified permeability changes, confirming barrier integrity in non-treated apical-out colonoids compared to EDTA-treated ones, but it did not directly verify fully intact tight junctions. In future studies, membrane integrity could be more directly evaluated in monolayer cultures, where the flat, single-cell layer structure enables more precise assessment of tight junctions and barrier function (67).

This study stably obtained apical-out canine colonoids and observed significant gene expression differentiation using a differentiation medium. Furthermore, these colonoids maintained their barrier integrity and could replicate host-pathogen interaction. Differentiated apical-out canine colonoids will accelerate both canine and human gastrointestinal research, enhancing our understanding of gastrointestinal physiology and pathology and helping the development of novel therapeutics.

## Data Availability

The original contributions presented in the study are included in the article/Supplementary material, further inquiries can be directed to the corresponding author.
