# Supplementary material for: Bacterial attachment and junctional transport function in induced apical-out polarized and differentiated canine intestinal organoids
Source: Front Vet Sci. 2024 Dec 18;11:1483421. doi: 10.3389/fvets.2024.1483421 (PMC11688377; doi:10.3389/fvets.2024.1483421)
Supplement: Supplementary file 1 [file Data_Sheet_1.docx]

Supplementary Material

**Supplementary Table 1.** Signalments of healthy dogs.

| **Dog** | **Breed** | **Sex** | **Age** |
| --- | --- | --- | --- |
| 1 | Boston Terrier | Spayed Female | 11 years old |
| 2 | Mixed Breed Dog | Castrated Male | 1 year old |
| 3 | Australian Cattle Dog | Castrated male | 7 years old |

**Supplementary Table 2.** Primers used in RT-qPCR.

| **Gene Name** | **Usage** | **Source** | **Forward Primer** | **Reverse Primer** |
| --- | --- | --- | --- | --- |
| SDHA | Internal reference | Peters 2007 | GCC TTG GAT CTC TTG ATG GA | TTC TTG GCT CTT ATG CGA TG |
| HMBS | Internal reference | Peters 2007 | TCACCATCGGAGCCATCT | GTTCCCACCACGCTCTTCT |
| LGR5 | Stem cell marker | Bongiovanni 2020 | GGCTCCACAGCCT AGAGACTTTAG | TTGTTGCTGTGAAATC CTAGTTCTTT |
| MUC2 | Goblet cell marker | Kramer 2020 | TCCTCTACCCTCGTCTACTGC | GACGGGCATGACCAGTTGAA |
| CHGA | Enteroendocrine cell marker | Kramer 2020 | GGTCCTGGCTCTGTTGTTCC | CTCGCGAGAAAAGACAACCG |
| ALPI | Intestinal epithelial cell marker | Sahoo 2023 | CGTAGTAAACCGCAACTGG | GGAAACATGTACTTTCGGC |

**Supplementary Table 3.** Composition of culture media used in this study.

| **Reagent** | **Expansion Medium (this study)** | **Differentiation Medium (this study)** | **Growth Medium (Csukovich 2023)** |
| --- | --- | --- | --- |
| Basal Medium | + | + | + |
| Noggin | 10% v/v (100 ng/mL) | 10% v/v (100 ng/mL) | 100 ng/mL |
| R-Spondin-1 | 20% v/v | 20% v/v | 10% v/v |
| Wnt3a | 100 ng/mL | − | 50% v/v |
| Primocin | 100 μg/mL | 100 μg/mL | − |
| N2 | 1x | 1x | − |
| B27 | 1x | 1x | 1x |
| Nicotinamide | 10mM | − | − |
| N-Acetyl-L-cysteine | 1mM | 1mM | 1 mM |
| EGF | 50 ng/mL | 50 ng/mL | 50 ng/mL (48h) |
| SB202190 | 10μM | − | − |
| A-83-01 | 500 nM | 500 nM | 500 nM |
| Gastrin | 10 nM | 10 nM | 10 nM |
| HGF | − | − | 50 ng/mL |
| IGF1 | − | − | 100 ng/mL |
| FGF2 | − | − | 50 ng/mL |

**Supplementary Figure 1.** The percentages of the fluorescence-positive cells in Apical-DM. The number of CHGA and SNA-positive cells was normalized by the total number of nuclei (DAPI). Data is shown as mean ± SEM. For image acquisition, randomly selected 4 fields were used in each biological replicate.

**
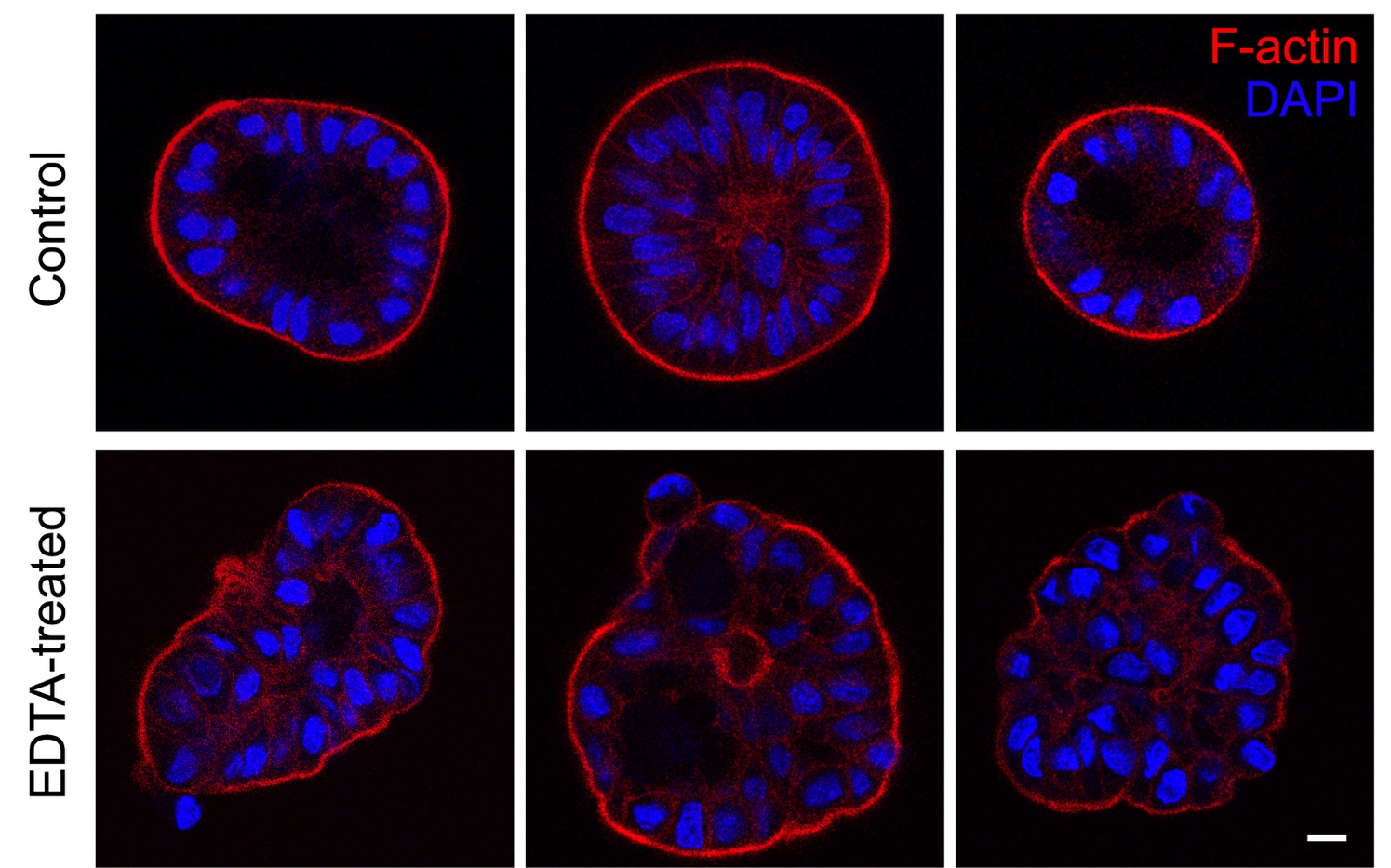
**

**Supplementary Figure 2.** Confocal microscopy images of control and EDTA-treated Apical-DM. Scale bar = 10 μm.


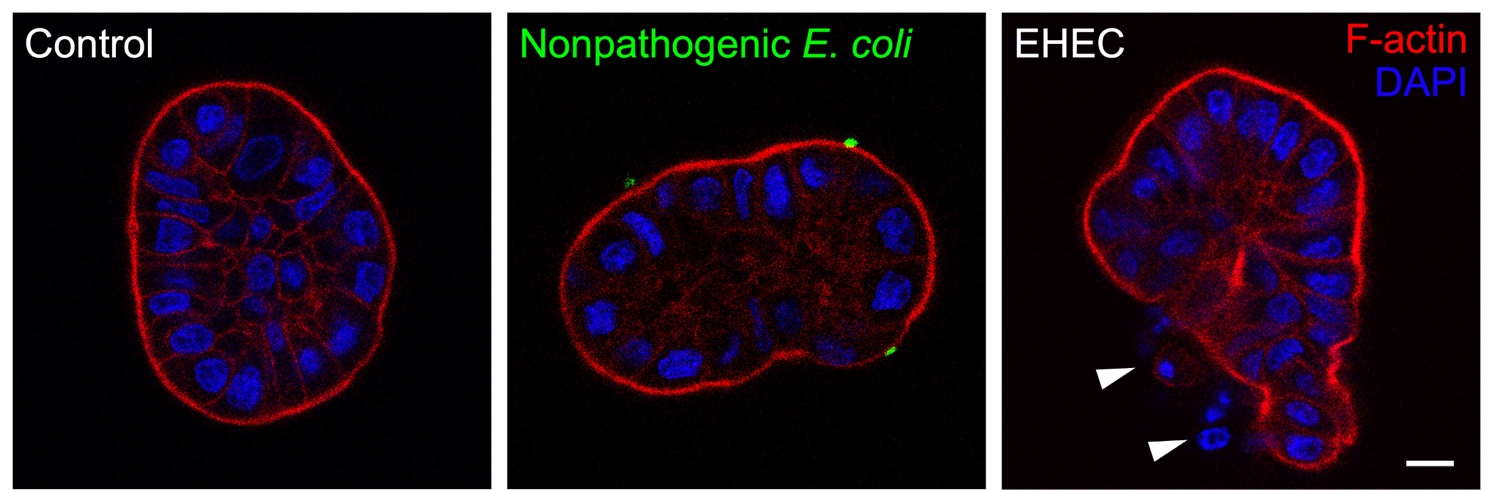


**Supplementary Figure 3.** Confocal microscopy images of control, nonpathogenic *E.coli* (YFP-tagged, green)-infected, and EHEC-infected Apical-DM. Scale bar = 10 μm.
